# Supplementary material for: Roles of IL-7R Induced by Interactions between Cancer Cells and Macrophages in the Progression of Esophageal Squamous Cell Carcinoma
Source: Cancers (Basel). 2023 Jan 6;15(2):394. doi: 10.3390/cancers15020394 (PMC9856499; doi:10.3390/cancers15020394)
Supplement: Supplementary file 1 [file cancers-15-00394-s001.zip › cancers-2134833-supplementary.pdf]

Supplementary Materials

Roles of IL-7R Induced by Interactions between Cancer Cells and Macrophages in the Progression of Esophageal Squamous Cell Carcinoma

Yu Kitamura, Yu-ichiro Koma, Kohei Tanigawa, Shuichi Tsukamoto, Yuki Azumi, Shoji Miyako, Satoshi Urakami, Takayuki Kodama, Mari Nishio, Manabu Shigeoka, Yoshihiro Kakeji, and Hiroshi Yokozaki

Table S1. Upregulated and downregulated interleukin-related genes in TE-11 cells co-cultured with macrophages.

Accession number	Gene title	Symbol	Log2 ratio (TE-11 co / TE-11 mono)
Upregulated genes (log2 ratio > 1.5)			
NR_120485.1	interleukin 7 receptor	*IL7R*	3.31
NM_001560.2	interleukin 13 receptor subunit alpha 1	*IL13RA1*	1.63
NR_048564.1	interleukin 1 receptor type 2	*IL1R2*	1.60
NR_052010.1	interleukin 11 receptor subunit alpha	*IL11RA*	1.51
Downregulated genes (log2 ratio < −1.5)			
NM_000576.2	interleukin 1 beta	*IL1B*	−1.73

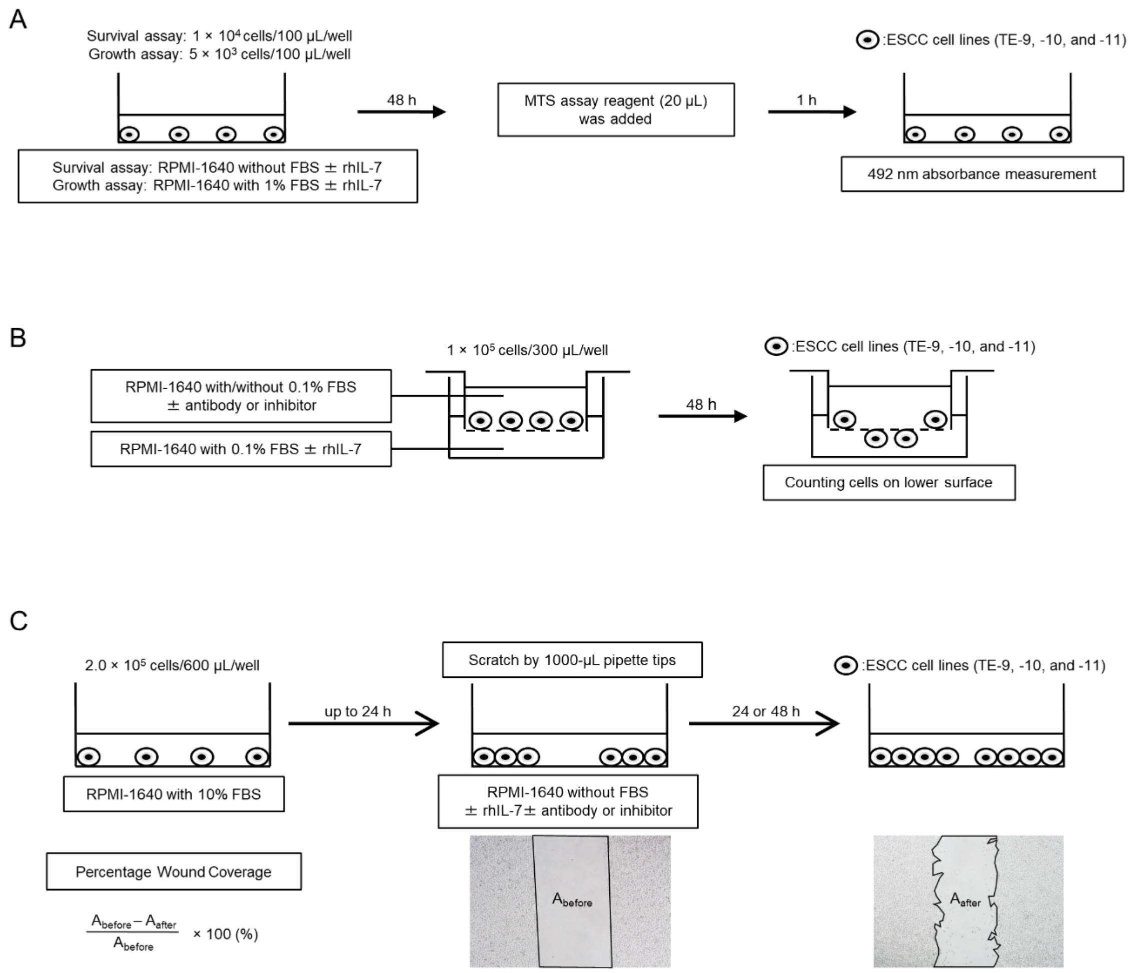

Figure S1: Schema of the experimental methods in cell proliferation assay (**A**), transwell migration assay (**B**), and wound healing assay (**C**).

Negative control (400×)

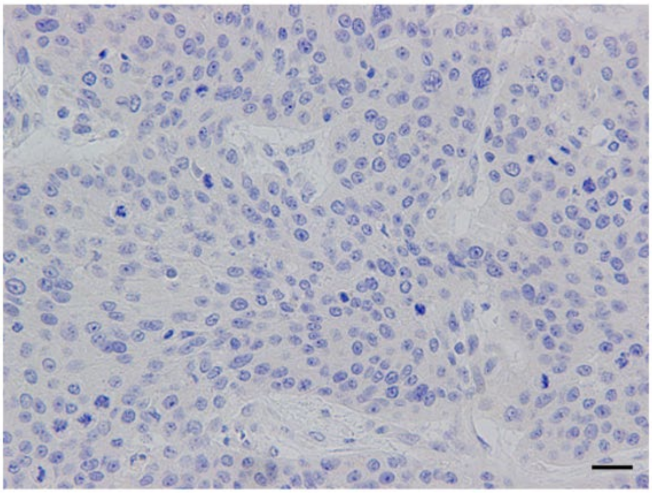

Figure S2. Negative control for immunohistochemistry in ESCC tissues. Immunohistochemical staining of ESCC tissue samples was performed using normal rabbit IgG as the negative control. The magnification of the picture is 400×, scale bar = 20 μm.

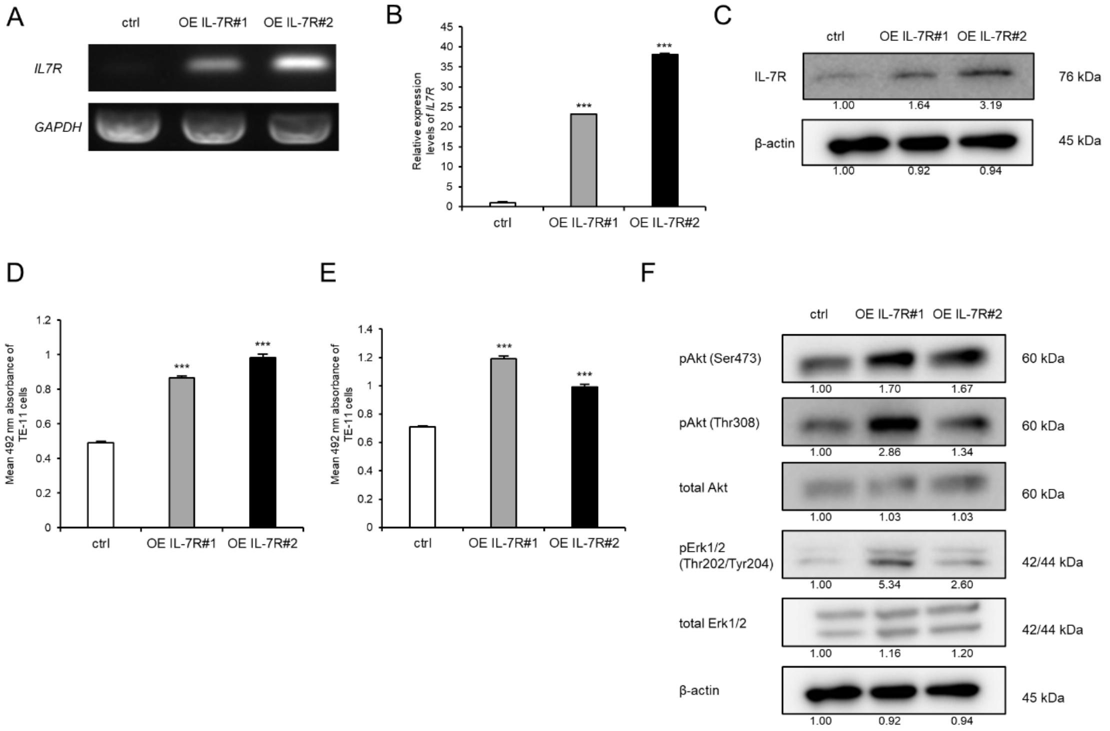

Figure S3. IL-7R overexpression promotes TE-11 ESCC cell survival and growth. (**A**, **B**) Expression levels of *IL7R* mRNA in TE-11 cells transfected with the control vector (ctrl) and IL-7R expression vector (OE IL-7R) were confirmed by RT-PCR (**A**) and qRT-PCR (**B**). *GAPDH* was used as an internal control. (**C**) Western blot analyses confirmed the expression of IL-7R protein in ctrl- and OE IL-7R-transfected TE-11 cells. β-actin was used as an internal control. The expression level was normalized using ImageJ software, and the relative value was set as 1.00 for ctrl-transfected cells. (**D**, **E**) Overexpression of IL-7R induces the survival (**D**) and growth (**E**) of TE-11 cells. (**F**) Western blot analyses demonstrated that the phosphorylation of Akt and Erk1/2 was induced by the overexpression of IL-7R in TE-11 cells. β-actin was used as an internal control. The expression level was normalized using ImageJ software, and the relative value was set as 1.00 for ctrl-transfected cells. Data are expressed as the mean ± SEM. *** $p < 0.001$.

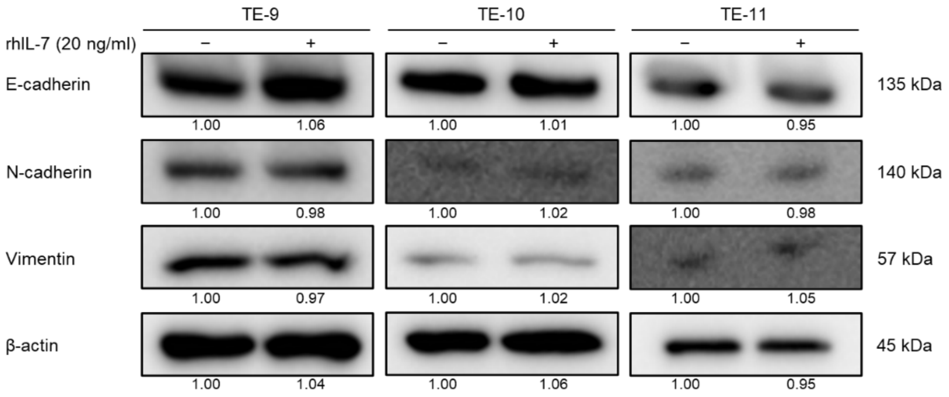

Figure S4. IL-7 does not affect epithelial-mesenchymal transition (EMT) in ESCC cells. To analyze the association between IL-7 and EMT-related factors, proteins were extracted from TE-9, -10, and -11 cells treated with/without recombinant human IL-7 (rhIL-7; 20 ng/mL) for 48 h. We confirmed the expression of the epithelial marker E-cadherin and the mesenchymal markers N-cadherin and vimentin using Western blotting. β-actin was used as an internal control. The expression level was normalized using ImageJ software, and the relative value was set as 1.00 for rhIL-7-untreated cells.

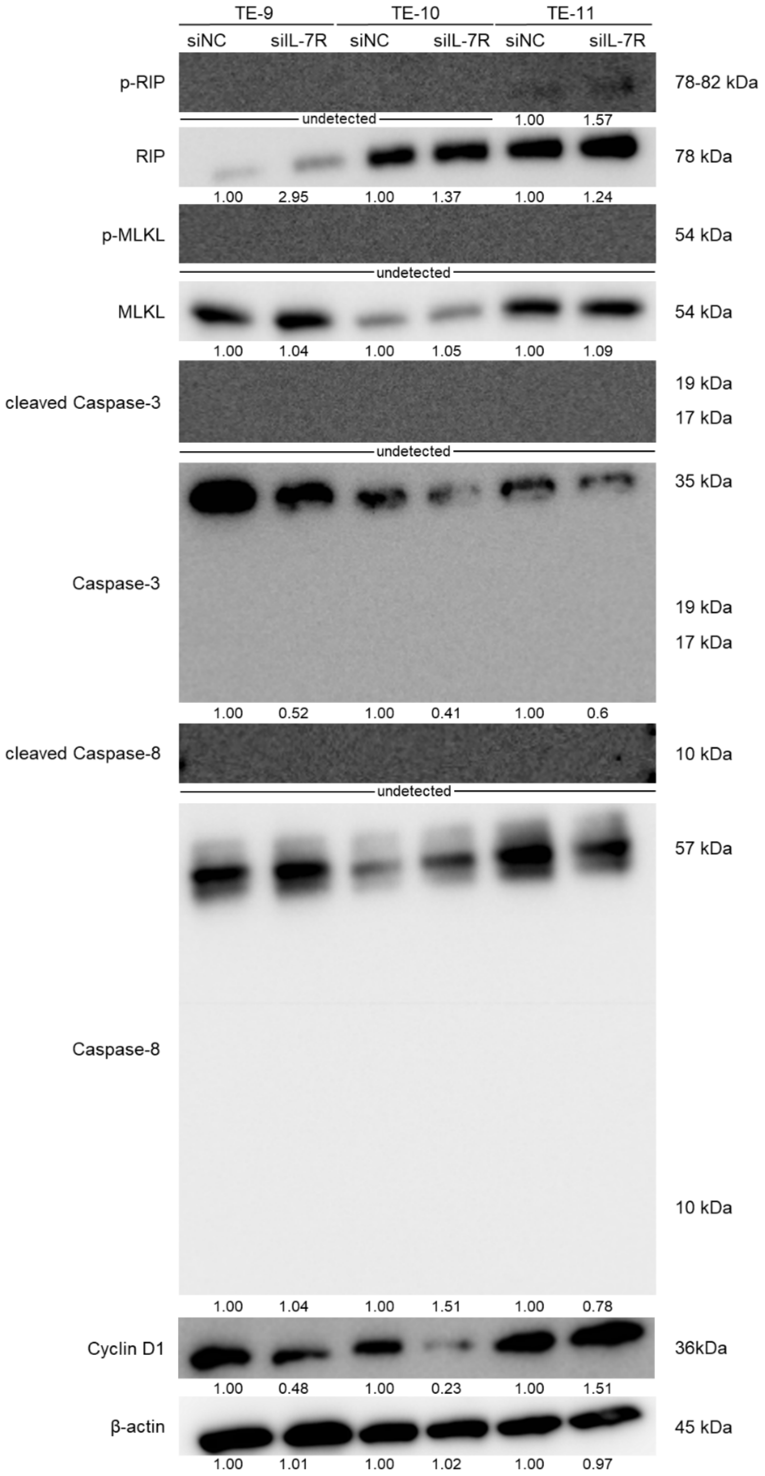

Figure S5. Expression levels of cell death- and cell cycle-related proteins in IL-7R-silencing ESCC cells. The expression levels of cell death-related proteins pRIP, RIP, pMLKL, MLKL, cleaved Caspase-3, Caspase-3, cleaved Caspase-8, and Caspase-8 and the cell cycle-related protein Cyclin D1 in TE-9, -10, and -11 cells transfected with siIL-7R or siNC were confirmed by Western blotting. β-actin was used as an internal control. The expression level was normalized using ImageJ software, and the relative value was set as 1.00 for siNC-transfected cells.

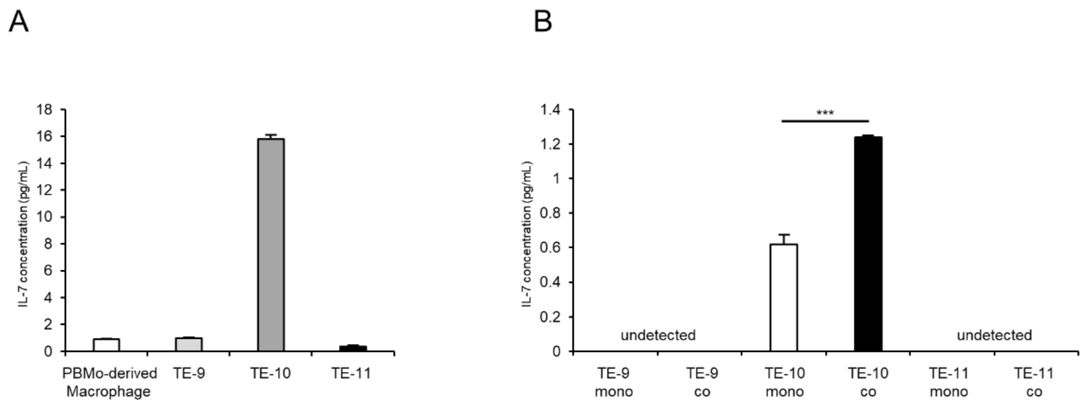

Figure S6. (**A**) Secretory concentrations of IL-7 in macrophages, TE-9, -10, and -11 cells were compared using ELISA. (**B**) Secretory concentrations of IL-7 in monocultured or co-cultured TE-9, -10, and -11 cells were compared using ELISA. Data are expressed as the mean ± SEM. *** $p < 0.001$.

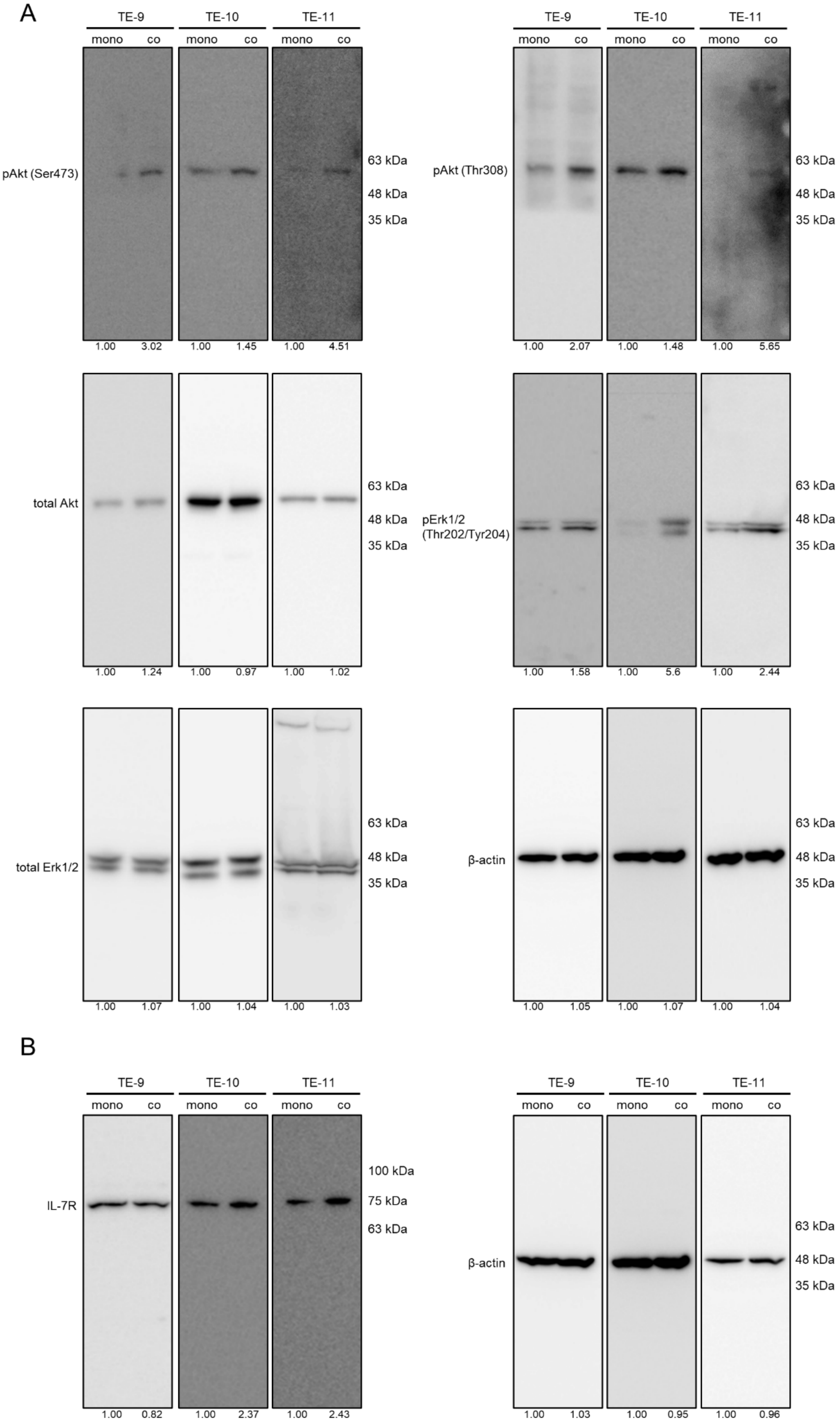
A
TE-9
TE-10
TE-11
mono
co
pAkt (Ser473)
63 kDa
48 kDa
35 kDa
1.00 3.02 1.00 1.45 1.00 4.51
pAkt (Thr308)
1.00 2.07 1.00 1.48 1.00 5.65
total Akt
1.00 1.24 1.00 0.97 1.00 1.02
pErk1/2
(Thr202/Tyr204)
1.00 1.58 1.00 5.6 1.00 2.44
total Erk1/2
1.00 1.07 1.00 1.04 1.00 1.03
β-actin
1.00 1.05 1.00 1.07 1.00 1.04
B
IL-7R
100 kDa
75 kDa
63 kDa
1.00 0.82 1.00 2.37 1.00 2.43
β-actin
1.00 1.03 1.00 0.95 1.00 0.96

C

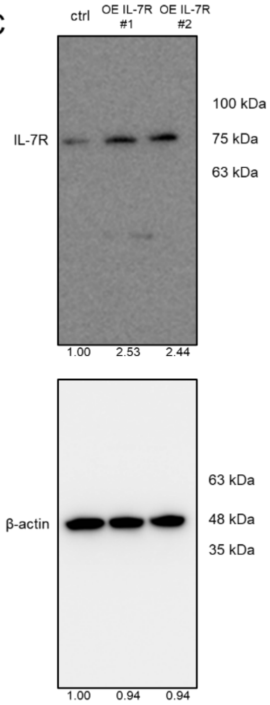
ctrl
OE IL-7R #1
OE IL-7R #2
IL-7R
100 kDa
75 kDa
63 kDa
1.00 2.53 2.44
β-actin
63 kDa
48 kDa
35 kDa
1.00 0.94 0.94

D

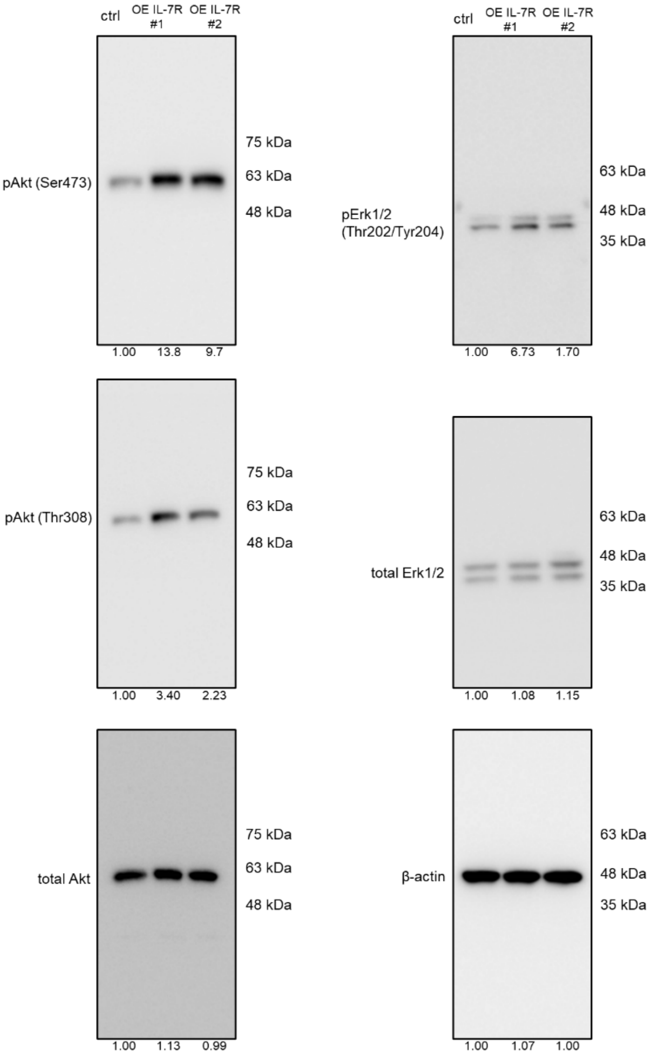
ctrl
OE IL-7R #1
OE IL-7R #2
pAkt (Ser473)
75 kDa
63 kDa
48 kDa
1.00 13.8 9.7
pAkt (Thr308)
75 kDa
63 kDa
48 kDa
1.00 3.40 2.23
total Akt
75 kDa
63 kDa
48 kDa
1.00 1.13 0.99
ctrl
OE IL-7R #1
OE IL-7R #2
pErk1/2 (Thr202/Tyr204)
63 kDa
48 kDa
35 kDa
1.00 6.73 1.70
total Erk1/2
63 kDa
48 kDa
35 kDa
1.00 1.08 1.15
β-actin
63 kDa
48 kDa
35 kDa
1.00 1.07 1.00

E

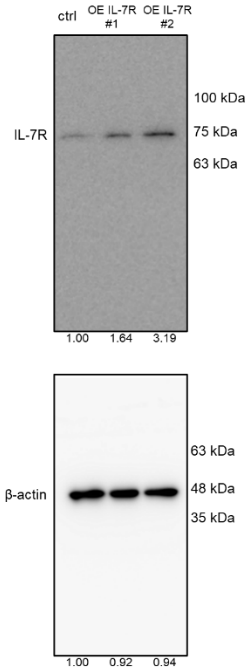
ctrl
OE IL-7R #1
OE IL-7R #2
IL-7R
100 kDa
75 kDa
63 kDa
1.00
1.64
3.19
β-actin
63 kDa
48 kDa
35 kDa
1.00
0.92
0.94

F

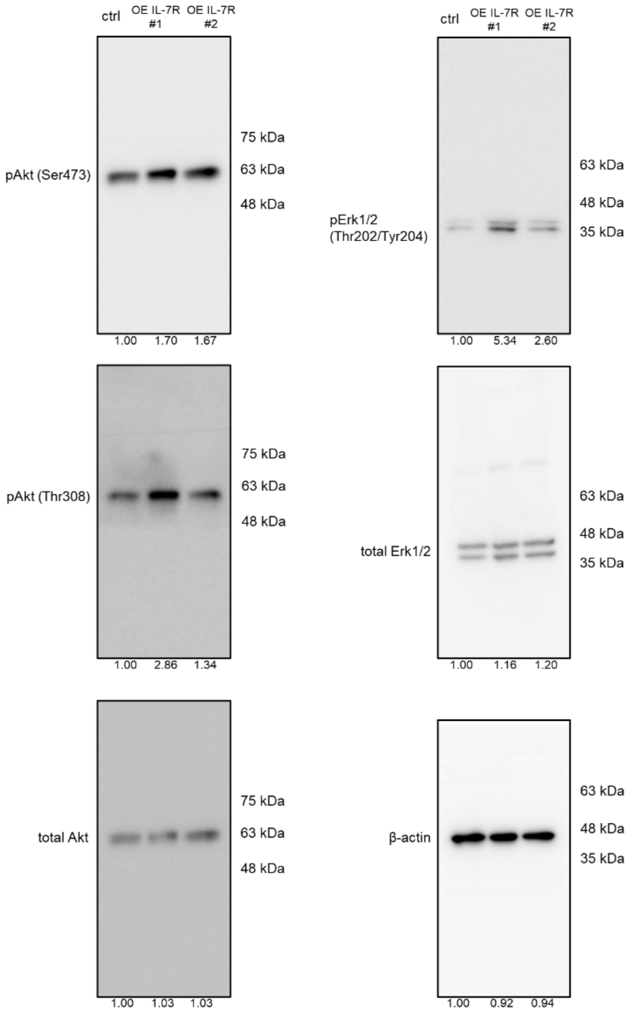
ctrl
OE IL-7R #1
OE IL-7R #2
pAkt (Ser473)
75 kDa
63 kDa
48 kDa
1.00
1.70
1.67
pAkt (Thr308)
75 kDa
63 kDa
48 kDa
1.00
2.86
1.34
total Akt
75 kDa
63 kDa
48 kDa
1.00
1.03
1.03
ctrl
OE IL-7R #1
OE IL-7R #2
pErk1/2 (Thr202/Tyr204)
63 kDa
48 kDa
35 kDa
1.00
5.34
2.60
total Erk1/2
63 kDa
48 kDa
35 kDa
1.00
1.16
1.20
β-actin
63 kDa
48 kDa
35 kDa
1.00
0.92
0.94

G

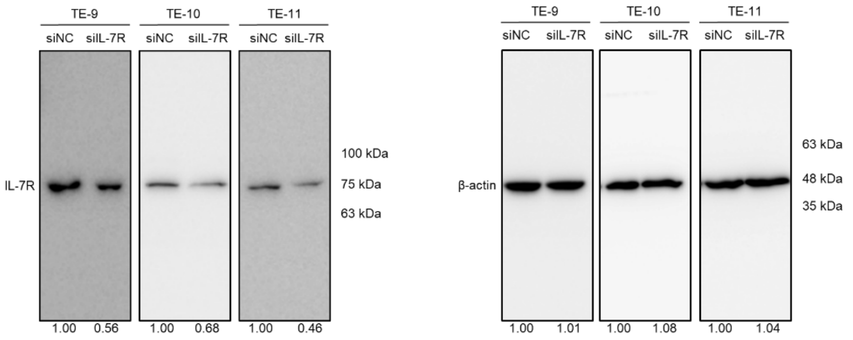
TE-9
TE-10
TE-11
siNC siIL-7R
siNC siIL-7R
siNC siIL-7R
IL-7R
100 kDa
75 kDa
63 kDa
1.00 0.56
1.00 0.68
1.00 0.46
β-actin
63 kDa
48 kDa
35 kDa
1.00 1.01
1.00 1.08
1.00 1.04

H

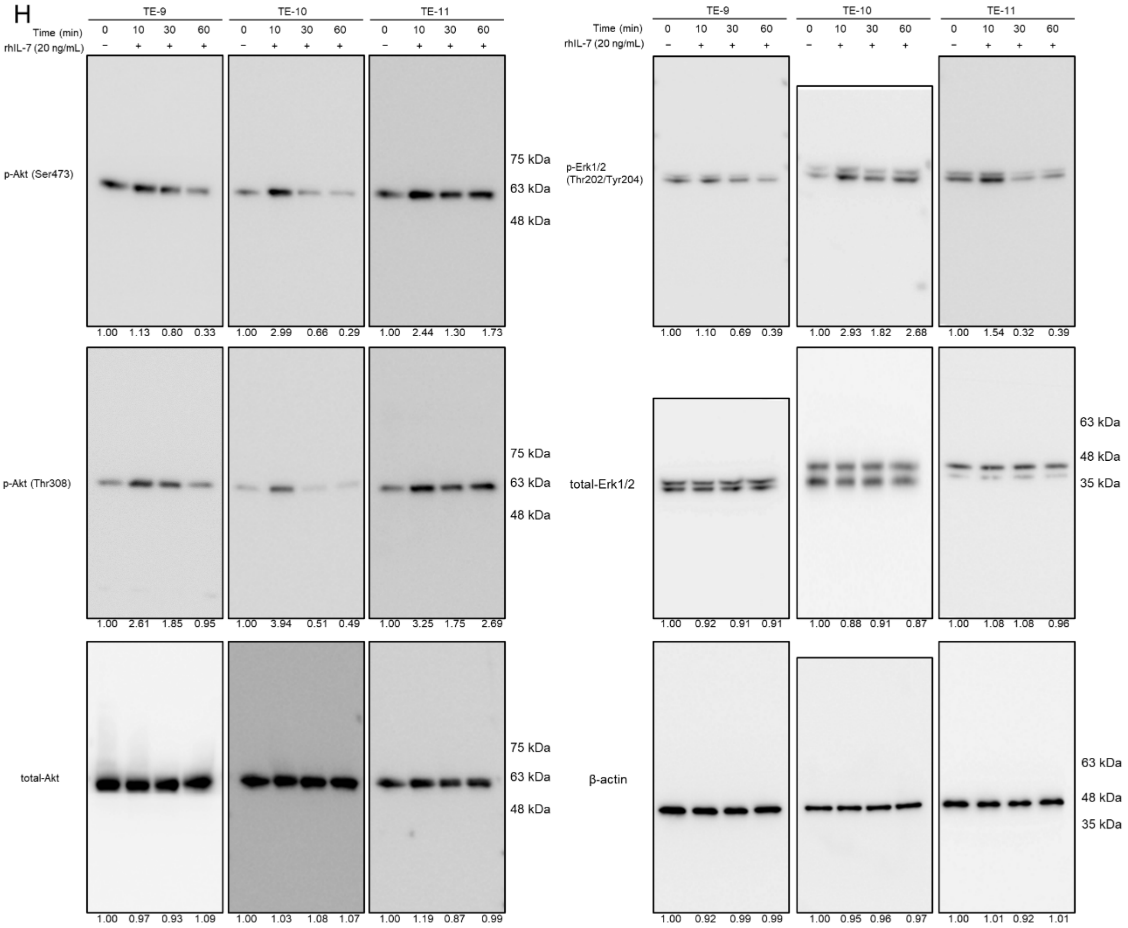
TE-9
TE-10
TE-11
Time (min)
0 10 30 60
rhIL-7 (20 ng/mL)
– + + +
p-Akt (Ser473)
75 kDa
63 kDa
48 kDa
1.00 1.13 0.80 0.33
1.00 2.99 0.66 0.29
1.00 2.44 1.30 1.73
p-Akt (Thr308)
1.00 2.61 1.85 0.95
1.00 3.94 0.51 0.49
1.00 3.25 1.75 2.69
total-Akt
1.00 0.97 0.93 1.09
1.00 1.03 1.08 1.07
1.00 1.19 0.87 0.99
p-Erk1/2
(Thr202/Tyr204)
1.00 1.10 0.69 0.39
1.00 2.93 1.82 2.68
1.00 1.54 0.32 0.39
total-Erk1/2
63 kDa
48 kDa
35 kDa
1.00 0.92 0.91 0.91
1.00 0.88 0.91 0.87
1.00 1.08 1.08 0.96
β-actin
1.00 0.92 0.99 0.99
1.00 0.95 0.96 0.97
1.00 1.01 0.92 1.01

I

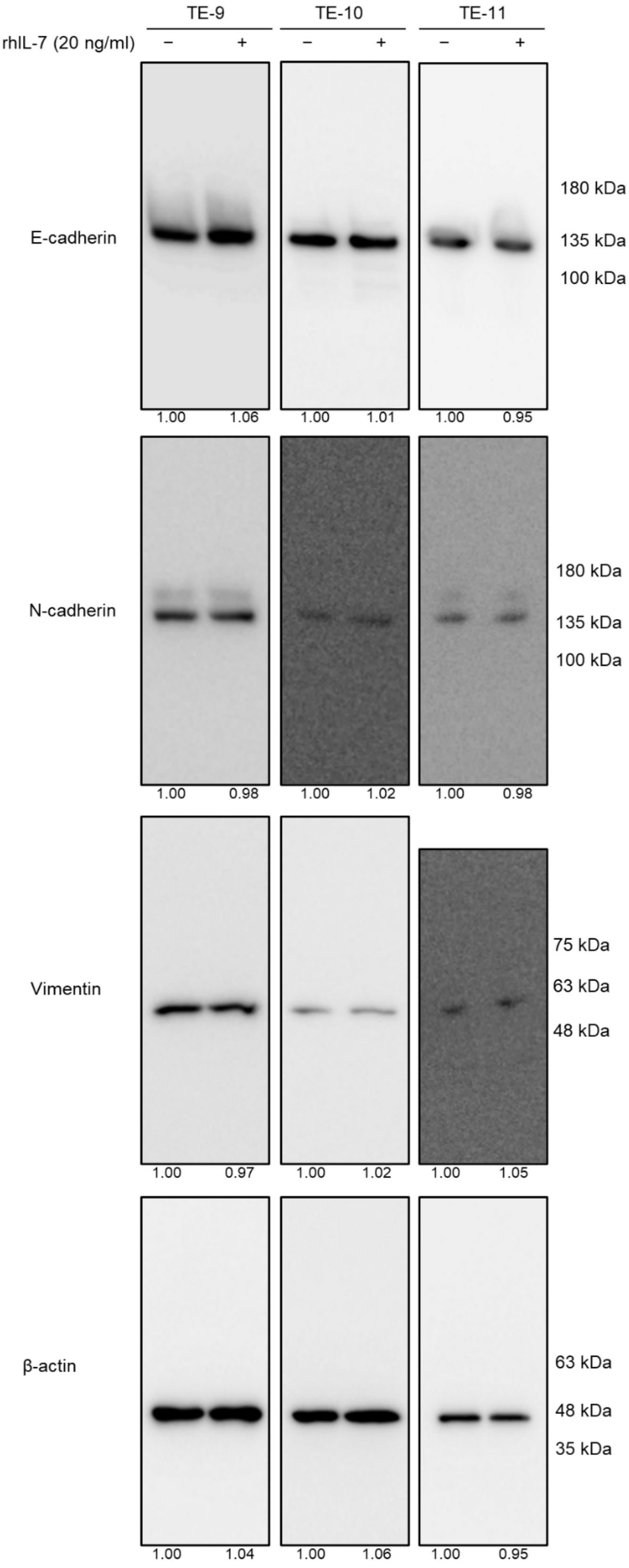
TE-9
TE-10
TE-11
rhIL-7 (20 ng/ml)
−
+
E-cadherin
180 kDa
135 kDa
100 kDa
1.00 1.06 1.00 1.01 1.00 0.95
N-cadherin
180 kDa
135 kDa
100 kDa
1.00 0.98 1.00 1.02 1.00 0.98
Vimentin
75 kDa
63 kDa
48 kDa
1.00 0.97 1.00 1.02 1.00 1.05
β-actin
63 kDa
48 kDa
35 kDa
1.00 1.04 1.00 1.06 1.00 0.95

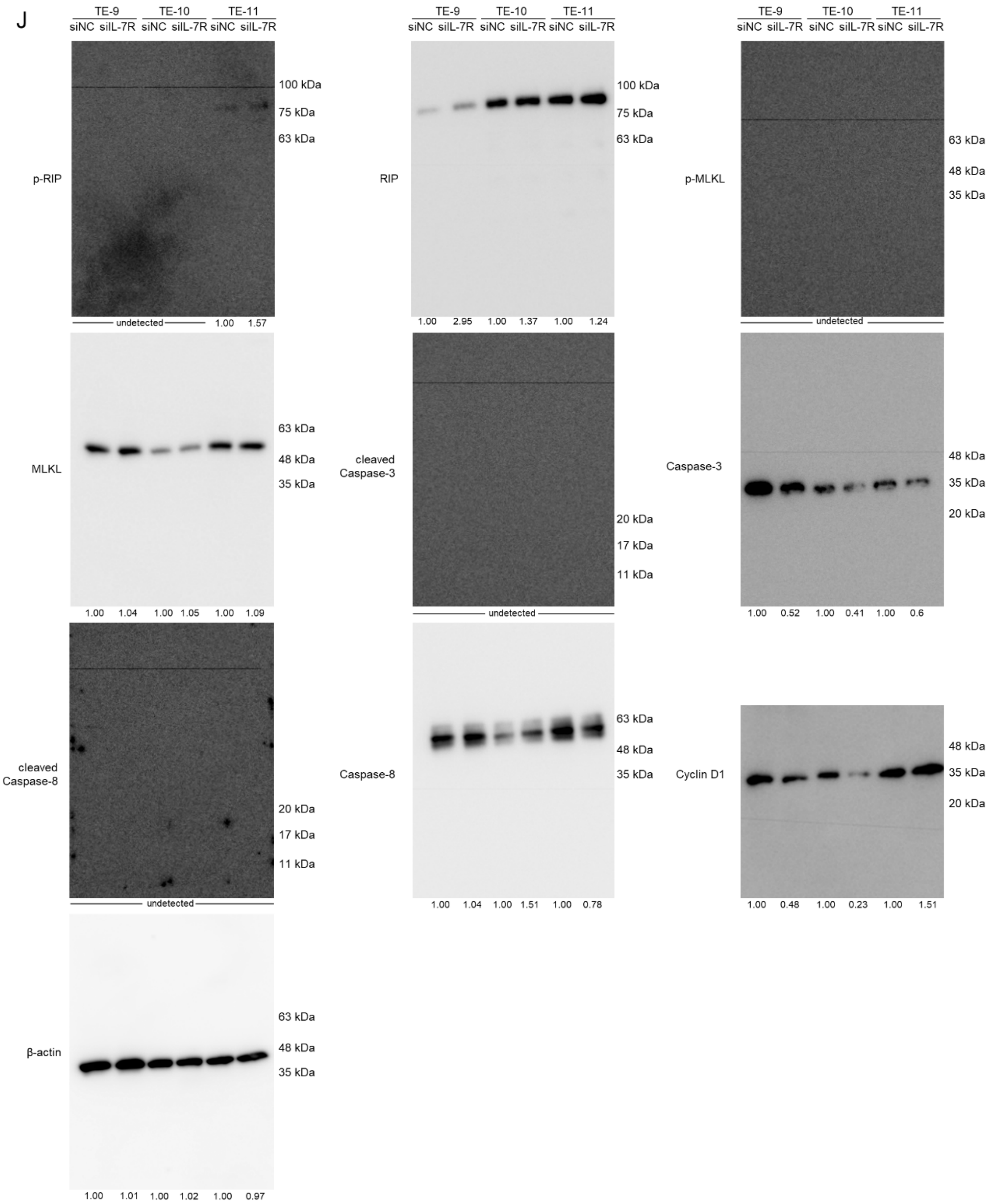

Figure S7. Raw image of Western blotting from Figures 1C, 1E, 2C, 2F, 3C, 5A, S3C, S3F, S4, and S5, corresponding to Figures S7A–J, respectively. The markers could not be visualized in the raw membrane data because three colored pre-stained markers that did not contain chemiluminescent substances were used.
